# Communication Abilities of Children with DoC after Severe Brain Injury in ICF Frames

**DOI:** 10.3390/ijerph18084267

**Published:** 2021-04-17

**Authors:** Anna Rasmus, Edyta Orłowska

**Affiliations:** 1 Institute of Psychology, Uniwersytet Kazimierza Wielkiego (UKW), 85-064 Bydgoszcz, Poland; 2 Institute of Psychology, Uniwersytet Gdański (UG), 80-309 Gdańsk, Poland; psyeo@ug.edu.pl

**Keywords:** functional diagnosis, ICF, minimally conscious state (MCS), TBI, communication

## Abstract

Introduction: The ability to communicate is one of the fundamental factors underlying human relationships. Severe brain damage and disorders of consciousness may indispose a person to participate in everyday social and family life. In spite of this fact, however, the issue of holistic approach to communication in the context of severe traumatic brain injury is still not well explained and described. The goal of this article is to introduce a profile of nonverbal behavior of children with disorders of consciousness. Materials and methods: The study included 30 children with minimal conscious state after severe brain trauma, aged between 7 and 16 years old. Research was conducted using the Coma Recovery Scale—Revised and the Bykova–Lukyanov Scale of Communication Activity. Results: Significant differences in communication level between investigated groups were demonstrated, both in Body Function (F = 9.184; *p* < 0.001) and Activity and Participation (F = 13.100; *p* < 0.001). Conclusions: It is possible to map and classify communication ability of children with minimal conscious state by using International Classification of Functioning, Disability and Health (ICF) protocol and the Bykova–Lukyanov Scale of Communication Activity, with specific consideration of Activities and Participation factors. This approach reveals differences in communication and disability level between children with minimal conscious state plus (MSC+) and minimal conscious state minus (MSC−).

## 1. Introduction

The ability to communicate is one of the most complex processes ever observed among living organisms [1]. At the same time, it is a common need of a person. It allows us to form relationships, socialize and generally interact with our surroundings. Human communication involves various skills and activities that contribute to general ability to exchange information between people. It can involve both linguistic and non-linguistic processes [2]. Communicating always takes place in certain environments that can be defined by a culture, a situation or a certain place. Ability to communicate is influenced by many factors. One of them is general health or, more precisely, body functioning. Amongst many conditions rapidly violating communication on many levels is traumatic brain injury (TBI). Data show that prevalence rate of TBI far exceed incidence rate of stroke or epilepsy. TBI occurs very often in children under 5 years of age, teenagers and young people between 15 to 24 years old [3] and often cause disorders of consciousness. Disorders of Consciousness (DoC) is a term used to describe a group of people suffering from severe brain trauma, who are waking up from a coma. This general category can be further separated into four states, considering the level of returning awareness, based on Recovery Coma Scale Revised: unresponsive state of coma (URS), minimal conscious state (MSC) and emergent conscious state (EMCS). The borders between those states are still a subject of discussion. New tools allow for even more accurate evaluation of actual functioning level of a patient. In this study, we focused on a group of children with MSC. People with this condition show minimal yet definite signs of consciousness, such as visual pursuit or command following but do not show functional communication. International Classification of Diseases for Mortality and Morbidity (ICD-11, 11th Revision, v2020-09) can be further divided into two consciousness states:

- Minimally conscious state plus (MCS+, 8E22.0)—typical for patients in a minimally conscious state who show signs of command following.
- Minimally conscious state minus (MCS−, 8E22.1)—describing patients in a minimally conscious state who show signs of non-reflex behavior (eye tracking, orientation to pain or contingent responses to specific emotional stimuli) but without command following.

It should be emphasized that a person in minimal conscious state (MCS) is indisposed in aspect of both perception and generating messages [4,5]. This state is difficult to assess using traditional tools, as impairment-based cognitive evaluations do not capture fully the complex communication problems of people with TBI in general [6].

In contrast to this approach, an assessment applying International Classification of Functioning, Disability and Health (ICF) produced positive results in this area [7,8]. Additionally, it is useful for practitioners [3], as it allows for considering communication along a continuum. It accommodates impairments of body structures and functions “representing the basic underpinnings of a communication disorder that influence individuals’ abilities to engage in functional communication activities and to participate in society” [9]. ICF was created in order to unify methodology and terminology of academic research on health, particularly health related states, results and indicators [10]. It was built upon a biopsychosocial model and, as such, integrates medical and social approaches formally used to describe disability and health and has great ecological value [11,12]. The ICF includes two basic parts: (1) functioning and disability and (2) contextual factors. Both of them are categorized into two components. Functioning and disability includes (a) body structure and function, and (b) activities and participation. The second component named as contextual factors involves (a) environmental factors and (b) personal factors. The ICF framework introduces human health concept as interaction on three levels: body part/body function (body structure and function), person (activity) and person in a societal role (participation). It is possible to describe the individual level of functioning by analyzing the interactions among environmental and personal factors and the components of body structure and function and activity and participation. In consequence, both ICF and ICD models complete and complement each other and give a holistic overview of a person’s functioning including strong points and deficits, as well as possible restrictions. Presently it is advised to use both knowledge based on medical diagnosis and functional descriptions concerning a patient [1].

The number of standardized methods of communication assessment for adults with severe brain injury, as well as children with developmental communication problems, is large. Only few amongst those include functional communication regarding activity and participation [13]. What is more, there are few diagnostic tools that can be utilized to describe potential communication impairment of children with established communicational and linguistic competence but lost it due to TBI. As of today, the Elton Bryson Stephens Company (EBSCO) research base contains one fully standardized method designed for children with minimal conscious state [14]. Most clinical research dedicated to communication behaviors of children in this group is based on short screening scales evaluating mostly eye contract, reacting to sounds and short instructions execution [15]. After analyzing available publications in this field, only one tool evaluating communication activity of children in minimal conscious state was found: Bykova–Lukyanov Scale of Communication Activity (SCLB) [14].

In view of the above, the authors attempt to introduce a profile of nonverbal behavior of children with DoC and verify if the ICF biopsychosocial model can be used to unify and extend efforts to fully describe their functioning in this area. The main research purpose was to map a standardized communication questionnaire often used in MSC onto the WHO–ICF framework and evaluate its usefulness in differentiating between MSC plus and minus state. As this study is mostly explorative in nature, the authors state 3 research questions:

1. Is it possible to map the Bykova–Lukyanov Scale of Communication Activity (SCALB) onto WHO–ICF framework? Mapping is defined as identifying ICF components needed to describe communication impairment of children with MSC, using SCABL results to fill the Communication in DoC checklist created by authors.
2. Is the ICF framework useful in outlining significantly different communication profiles for MSC plus and minus underage patients?
3. What are the characteristics and level of communication disability for children with MSC?

## 2. Materials and Methods

### 2.1. Participants

The study was conducted in a specialized neurorehabilitation center Neuron in Bydgoszcz, Poland, and involved 30 children with severe TBI (12 girls and 18 boys), between 7 and 16 years old. The mean age was 11 years ± 2.3 years old. Eligibility criteria included the following: minimal consciousness state plus or minus (MSC + or −) as confirmed in Coma Recovery Scale-Revised (CRS-R) and parent’s/legal guardian’s consent to participate.

The study protocol excluded children with chronical vegetative or unresponsive state, as confirmed in CRS-R, and those able to communicate their needs verbally or using an eye-tracker. Other exclusion criteria were defined as follows: patients diagnosed with autism, cerebral palsy, severe visual and hearing impairment were eliminated from the study, as well as children with previous neurological or intellectual problems. Lack of potential subjects’ parents or legal guardians’ full consent also was part of exclusion criteria.

### 2.2. Study Design

As MCS is a relatively rare and clinical state randomization was not possible, this study was designed by using purposeful selection of research sample. All the children met all the inclusion criteria mentioned above. Assessment was conducted between 2018 and 2019.

### 2.3. Study Protocol

The protocol of this study was approved by the local Bioethics Commission of the CM UMK, Toruń, Poland (KB 546/2018). Informed consent for the children’s participation in the study was obtained in writing from their parents or legal guardians. Experimental conditions were in compliance with the Declaration of Helsinki. No adverse events were observed during the study. No intentional deviations from the protocol were observed during the study.

### 2.4. Measurements

The assessments were carried out once in rehabilitation center environment. The following research tools were used:

Coma Recovery Scale-Revised (CRS-R) [16]—standardized assessment measure of consciousness state. It was designed in order to capture discreet shifts in neurobehavioral status of people with DoC. It contains 6 subscales evaluating auditory, visual, motor, oromotor, communication and arousal processes. This scale includes 29 items with hierarchical organization. The reliability of the CRS-R in examining conscious awareness progress has been extensively demonstrated. Results in this scale significantly correlate with clinical outcome at discharge from medical facility. CRS-R scores proved to have excellent concurrent validity with other well-known neurobehavioral scales, such as the Glasgow Outcome Scale and the Disability Rating Scale [16].

The Bykova–Lukyanov Scale of Communication Activity (SCABL), [14] adapted by Pąchalska [17], is a tool widely used in communication assessment amongst DoC patients [14]. It involves observation and ranking a patient’s response according to a three-point assessment depending on the presence and frequency of communication signals:

- “0”—complete lack of communication signals,
- “1”—“unstable”—sporadic communication signals,
- “2”—complete recovery and restoration of communication signals.

The total summarized score is calculated and includes the total amount of signals in each separate section of the described systems. Those signals include the following: body responses, gestures, vegetative reactions, facial gesture, speech and contact level.

The questionnaire includes four negative statements and nine conditionally negative statements. Higher total score signifies a higher level of consciousness and the child’s ability to communicate with the outer world.

### 2.5. Procedure

The completion of the SCABL (104 parameters) was done by a medical psychologist.

The scoring guidelines and test forms of SCABL were used in the mapping procedure. Each questionnaire item was reviewed separately by 5 competent judges chosen from experts in the field of communication and brain trauma. Each item in the applied test was mapped onto the domains of the WHO–ICF framework. This included body structure and function, activities and participation. Processed items were further categorized or mapped onto the WHO–ICF components, following a protocol similar to that conducted by Ostensjo, Bjorbaekmo, Carlberg and Vollestad [18] in their ICF-based mapping procedure on the Pediatric Evaluation of Disability Inventory (PEDI). What is more, the definitions of each of the sections of the WHO–ICF and equivalent definitions of coded items (e.g., communication, speech, etc.) were employed in the charting procedure. In our study, utilized items were associated with only one of the WHO–ICF components based on mutual agreement by the competent judges and authors. Amongst available options introduced by WHO authors, in order to solve problems in matching test items with ICF components, the authors chose the one presenting activity and participation as uniform, overlapping components [10]. Table 1 presents the results of this mapping.

### 2.6. Statistical Analysis

Statistical analyses were performed using Statistica 13.1. software developed by StatSoft Polska. Between group comparison involved ANOVA and post hoc Turkey analyses. In order to analyze within-group comparisons of ICF subcategories, Student’s *t*-test for independent group was conducted.

## 3. Results

SCLB raw scores were grouped by ICF category by engaging the competent judges’ procedure. Five field specialists, with both clinical experience and theoretical knowledge on communication, gave their educated opinion on this issue. Psychologists and speech therapists alike judged Body Function (BF) and Activity and Participation (A&P) to be crucial areas.

As a next step, unified indicators were calculated for each of those ICF sections, with a maximal score of 100 points in each subscale. Table 2 presents the results of this procedure for Body Function (BF) and Activity and Participation (A&P).

**Figure 1:**
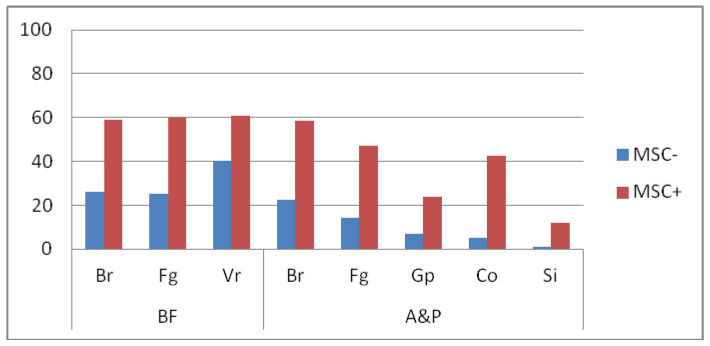
Differences between MSC+ and MSC− scores in specific ICF, BF and A&P subcategories of potential communication behaviors.

BF area was paired with chosen items of Body reaction (Br), Facial gestures (Fg) and Vegetative reaction (Vr) of SCLB. The A&P area was matched with Body Reaction (Br), Facial gesture (Fg), Gestures and pose (Gp), Contact with the outer world (Co) and Speech and intonation (Si) items. Comparisons between MSC− i MSC+ subjects’ performance according to this arrangement were made by using ANOVA and HDS Turkey post hoc test. The effect demonstrated in this analysis includes BF area matched with SCLB scores. Post hoc comparisons using the Turkey test indicated significant differences between groups in all three subcategories. MSC− patients’ average score in Br and Fg was 35 points lower than MSC+ subjects. In Vr, this difference was less apparent, being 20 points. Similarly, the effect for potentially performed communication behaviors was observed. MSC− patients scored significantly lower, which indicates less such behaviors in their repertoire when compared to MSC+. These differences between investigated groups are depicted in Figure 1.

Further analysis involved within-group comparisons of ICF subcategories, in order to show profile of potential communication behaviors presented by MSC− and MSC+ patients. The analysis of BF area of ICF showed significant difference only between FG and Vr in MSC− group (t = 2.071; *p* = 0.047). The MSC+ of the BF area of ICF showed no significant difference between scores of each subcategory. For the A&P area in the MSC− group, Br was significantly higher than Gp (t = 3.346; *p* = 0.002), Co (t = 3.618; *p* = 0.001) and Si (t = 5.251; *p* < 0.001). Fg was significantly higher than Co (t = 2.051; *p* = 0.039) and Si (t = 3.810; *p* = 0.001). Similarly, MSC+ scored higher in Br subcategory than in all other subclass except for Fg (*for Br vs. Gp:* t = 3.038; *p* = 0.005; for Br vs. *Co*: t = 3.015; *p* = 0.005; for Br vs. Si: t = 5.983, *p* < 0.001). Additionally, Fg seem to be more significant as MSC+ subjects score higher in this aspect when compared to Gp (t = 3.899; *p* = 0.001). Fg scores are also higher than Si (t = 6.826; *p* < 0.001). What is interesting, Co is evaluated as higher than Gp (t = 2.841; *p* = 0.009), which is the opposite result than in MSC−.

Finally, authors of this study made an attempt to summarize communication disability level in accordance with ICF guidelines. As mentioned above, all SCLB subscales were mapped with ICF matrix and divided between two areas (BF and A&P). As each subcategory was unified into a 100-point scale, a level of fulfilling it could be calculated and expressed in percentage. Gathered data were classified according to levels of disability introduced in ICF manual (Table 3):

- 0–4% (no problem present);
- 5–24% (mild problem);
- 25–49% (moderate problem);
- 50–95% (major problem);
- 96–100% (extremely large problem).

Our analysis indicates that MSC− patients reach higher disability level than MSC+ subjects in all investigated communication categories. Most of children with MSC− can be classified as having a major problem in all subcategories of BF. In A&P, major problems are most common in Br and Fg, while Gp scores usually point to major and extremely large communication disability. Co and Si are usually considered as extremely large difficulty in functioning.

MSC+ children are mostly included in the BF area of ICF as experiencing moderate problems. In the A&P category, they most commonly have moderate difficulties in functioning when Br and Fg subcategories are analyzed, while major difficulties in Gp, Co and Si.

## 4. Discussion

Awareness level and functioning of people in minimal conscious state are differentiated with the use of Recovery Scale-Revised or Brief Post-Coma Scale (BPCS) and Full Outline of Unresponsiveness Scale (FOUR). Most publications focus on distinguishing between unresponsive state of coma (URS) and minimal conscious state (MSC). Neuroimaging and functional assessments show distinct patterns among MCS patients and lead to separating out MSC− and MSC+ conditions.

This study’s goal was not to verify if those states can be differentiated clinically but how those two conditions differ according to ICF classification language. We were interested to know is it possible to distinguish between them by using this universal terminology describing specific communication activities performed by patients.

The need to unify communication disorders of people in minimal conscious state is prevalent, as this area of functioning is of interest to psychologists, speech-therapists as well as medical staff applying their own psychological, pedagogical and medical terms to the same condition or behavior. Up till now, the assessment of communication cited in publications involved mostly screening methods. Those tools, however useful, are based on stating if a person is able to comply with a command, keeps eye-contact and reacts to auditory stimuli, and they involve verbalization assessment. Such an assessment is sufficient to determine awareness level but far less helpful in therapy planning and evaluating its effect. In this case, a much more detailed appraisal of various communication activities is required. Such an approach can be essential for training and informing family members of people experiencing brain trauma in order to direct their attention onto potential communication behaviors, such as body signals, that can be interpreted and used to establish a better relationship with an underage patient.

The first aim of the current study was to assess usefulness of SCABL questionnaire in mapping disability and functioning of patients with disorders of consciousness using the ICF, which is based on the biopsychosocial model. The authors of this research were also interested in characterizing the level of communication disability for children with MSC and outlining communication profiles for MSC plus and minus underage patients. The Bykova–Lukyanov Scale of Communication Activity (SCABL) allows for detecting many and various signals from different channels (verbal and non-verbal) and can be considered as sensitive tool for diagnosing patients in a deeply altered state of consciousness (vegetative state, minimal conscious state and so on) [14].

As well as most impairment-based communication measures conventionally used by speech-language pathologists in their practice, it is easily available and can be administered scored and interpreted in relatively straightforward manner [6]. This assessment, nevertheless, does not offer a sufficient picture of the communication skills MCS children [3]. The difficulty level of allocating functional communication test items to each of the components and parts of the WHO–ICF framework and to the domains within these components reveals the intricacy of human communication and its crucial role in our every-day life.

Our findings demonstrate, however, that the SCABL questionnaire can be used to categorize functional communication to both activity and participation components of the WHO–ICF. As most researchers experienced difficulties resulting in assigning functional communication items from the tests to both Activity and Participation domains of the WHO–ICF, we decided to unify this area into one [3,18]. Both domains are crucial for clinical practice. The competent judges were asked to omit the structures domain, as it is of interest to doctors, physiotherapists rather than psychologists and speech therapists.

Secondly, the results of the comparison between mapped results of functional communication of children with MSC+ and MSC− show significant differences between them. MSC+ patients have generally better body functioning, including more harmonious profile between body reactions, facial expressions, and vegetative reactions. They tend to have a richer repertoire of motor behaviors and controlled body movements (mostly arms and legs) are more often observed among this group. This can be considered as a good base for rebuilding communication skills.

In contrast, the MSC− children body functioning is more diversified considering types of reactions, their stability and frequency. While most signals observed in this group include vegetative reactions, it is advised to be prudent and careful in their interpretation as potentially communicative in nature. Those signals can occur as dependent and independent depending on the specific situation. They can carry an information, but it is difficult to connect such a reaction with a specific answer to external stimuli. This corresponds with similar findings that reported high inconsistence in aware behaviors of MCS patients [19,20,21]. Low scores in body structure can hint to plan motor functions, strengthen orofacial muscles, teach basic gestures and work with a patients breathing control. This area rehabilitation seems to be of most importance for children with MSC−.

On Activity and Participation level comparison between MSC+ and MSC−, patients also show better functioning of the former. Children with MSC+ also showed varied communication oriented behaviors, with more widely spread stability and frequency, in which they occurred. Their face expression was more vivid, which facilitates message reception. They present higher levels of contact. They can be receptive to communication attempts and have higher levels of initializing communication. Among these subjects, increased motor activity was observed, which was categorized as a response to situation and presence of close relatives or strangers. This was not noticed among MSC− children, where passive reception is most common and reaction to clear and repeated pleas from their caregivers are rare and irregular.

Both MSC− and MSC+ children tend to score the lowest on activity and participation level in speech area. Amongst MSC+ subjects, vocalizations are present. They can call out by using a sound, such as coughing, to ask for attention. Occasionally the can say short words, such as “yes” or “no”, and participate in a simple dialog, using an established code or eye-tracking technique. Their conversation comes with great difficulties and is limited to closed questions.

These findings are in agreement with our previous research aiming to describe communication of patients with MSC [22,23].

Overall, research results reflect the usefulness of mapped ICF protocol in differentiating communication level of MSC underage patients. Therefore, we state that using domains of biopsychosocial model is a justified and beneficial approach to asses and understand functioning of children with MSC. It provides a way to unify research results by translating observations to internationally accepted framework. What is more, a functional approach can be applied to rehabilitation linking all areas of diagnosing and therapy theoretically, conceptually and clinically to communication problems considered as essential in MSC state [3,24,25,26].

Additionally, it demonstrates the fact that using psychological test concentrating purely on functional aspects can be insufficient. ICF framework offers division between body impairment and difficulties in applying various skills in real life. A person can have deficits in the body structure domain and therefore cannot participate in social life. However, one can also have no limitations concerning the body but experience motivational or executive difficulties, which give similar end result.

To conclude, all children with MSC experience disability in communication. The severity level varies, however, as MSC+ patients usually reach the level between moderate and severe. MSC+ group can be characterized as majorly disabled in speech, intonation and gestures using aspect but moderately disabled when body reactions or facial expression are taken into consideration. MSC− children experience major or extremely large disability. The absolute lack of functioning was observed in using gestures and speech or intonation, as well as general contact aspect. Major impairment and limitations in functioning were present also in Body Functions and Activity and Participation domains in body reaction and facial expression aspects. In addition to determining deficits and giving reference to disability level, the ICF classification allows us to name a person’s resources that can be built upon during the rehabilitation process. The strong points of children with MSC+ include eye contact, ability to visual fixation ability, showing unwillingness with facial expression or gesture, trying to move away from unpleasant stimuli and expressing suffering and frustration. Generally, they are able to efficiently communicate their emotional state.

Resources of MSC+ patients undeniably include compatibility of emotional and body reactions, as well as more adequate emotional expression and higher level of facial expression. Patients can make controlled body movements, which reoccur regularly and can be used in forming understandable messages. Better body control and acting along with instruction are more stable in time and allow for better cooperation with therapists. It facilitates introducing alternative communication forms while rebuilding verbal functions.

This study has obvious limitations, as it involved a small number of people, and trail randomization could not be applied. More research conducted according to the ICF framework is needed to validate our presented conclusion.

## 5. Conclusions

Admittedly, we stated three questions: (1) Is it possible to map the SCALB questionnaire onto the WHO–ICF framework? (2) Is the ICF framework useful in outlining significant differences for children with MCS? (3) What are the characteristics and level of communication disability for children with MCS?

It is possible to map and classify the communication ability of children with DoC by using the ICF protocol and SCABL, with specific consideration of Activities and Participation factors? This approach reveals differences in communication and disability level between children with MSC+ and MSC−. Communication activities on Body Function level are more common among children with MCS−. As such, this can be considered as the foundation for neuropsychological therapy planning centered on a patient’s resources. Among younger people with MCS+, active participation in communication acts is significantly higher and repeated nonverbal responses are more common, but the ability to communicate one’s needs is still lacking.

## Figures and Tables

**Table 1:** Results of applying the mapping procedure of the Bykova–Lukyanov Scale of Communication Activity (SCLB) to International Classification of Functioning, Disability and Health (ICF) components.

	Body Function	Activity and Participation
Body reaction	Acceleration–slowdown of breathing on request (1); finger movements (4); hand movements (6); involuntary body movements (13,15,16,17,18); tertiary bodily signals (36); involuntary eye opening (27).	Adjust breathing on command (2,3); move limbs on command (5,7); clench fists (10); handshake (11); purposefulness in body movements to be in contact (14); Changing the position of the body in response to contact (21); head turn to the direction of voice (25); head away from speaker (26); eyes opening as a response to conversation(28); and to deep contact (29); fixation (30); withdrawal from contact through eye abduction (31); expressing aggression with the body (33); expressing negativity with the body (34); voluntary “yes” response using eyes (38).
Facial gesture (Fg)	Non-differentiable facial gestures (39); facial paleness (40);blushes on cheeks (41); raising of the eyebrows (42); emotional lability (57).	Changes of eye expression during contacts (43); pain grimace (44); frustration (45); weeping (46); expression of insult (47); irritation (48); smile (49); laugh (50); fright, fear (51); disappointment (52); mimic reactions to relatives (55); adequacy of emotional expressions (56); connection emotions with the actual situation (58); understandable psychological emotions (59); recognition of other signals during repeated contacts (60).
Vegetative reactions (Vr)	Change in skin color (61,65,66,71,72); Change in temperature (62,64,69); Sweating (63); hyperkinesis (68); change in the pupil size (70); perspiration (67).	----
Gestures and pose (Gp)	----	Gestures while answering (74); gestures indication own intentions (75), change of body pose during contacts (76) crossed arms on the chest (77); crossed legs (78); closed pose (79), fear during body contacts (80); pose of contact desire (81).
Contact with the outer world (Co)	----	Adequate understanding of the fact of interaction with other person (82); contact with other (84, 85, 86); adequacy of contacts (87), depth of contacts (88); motivation to the emotions of others (90); sufficient involvement in contacts with others (90); the congruence of various own body signals when making contact with others (91); understanding humor (92).
Speech and intonation (Si)	----	Optimal speed of responses (93); regularity of answers (94); notional adequacy of responses (95); the congruence of verbal responses to nonverbal signals (96); tone coloring of speech (97); variability in speech volume (98), timbre variability (99); intonation (100); pauses (101); emotional adequacy in speech (102); adequacy of emotional expression in verbal responses (103); opportunity to talk (104).

**Table 2:** Differences between minimal conscious state minus (MSC−) and minimal conscious state plus(MSC+) patients’ potential communication behaviors corresponding with Body Function (BF) and Activity and Participation (A&P) areas of ICF.

ANOVA						Post Hoc Comparisons	
	df	Mean Square	F	*p*	Eta-Square	Between Group Comparisons	*p*
BF area	3	0.485	9.184	<0.001	0.514	BF_Br_ MSC− vs. MSC+	<0.001
						BF_Fg_ MSC− vs. MSC+	<0.001
						BF_ Vr_ MSC− vs. MSC+	0.029
A&P area	5	0.268	13.100	<0.001	0.732	A&P_Br_ MSC− vs. MSC+	<0.001
						A&P_Fg_ MSC− vs. MSC+	<0.001
						A&P_Gp_ MSC− vs. MSC+	<0.001
						A&P_Co_ MSC− vs. MSC+	<0.001
						A&P_Si_ MSC− vs. MSC+	<0.001

BF area, Body Function area; A&P area, Activity and Participation area; BF_Br, Body Function_Body reaction; BF_Fg, Body Function_ Facial gesture; BF_Vr, Body Function_ Vegetative reactions; A&P_ Br, Activity and Participation_ Body reaction; A&P_Fg, Activity and Participation_ Facial gesture; A&P_Gp, Activity and Participation_Gestures and pose; A&P_Co, Activity and Participation_Contact with the outer world; A&P_Si, Activity and Participation_ Speech and intonation.

**Table 3:** Percentage distribution of communication disability level in each BF and A&P subcategories amongst children with MSC− and MSC+**.**

			No Problem (%)	Mild (%)	Moderate (%)	Major (%)	Extremely Large (%)
BF	BF_Br_	MSC−	0	6.25	6.25	75	6.25
		MSC+	0	14.28	57.14	28.57	0
	BF_Fg_	MSC−	0	6.25	0	81.25	6.25
		MSC+	0	21.42	50	28.57	0
	BF_Vr_	MSC−	0	6.25	25	62.5	0
		MSC+	7.14	14.28	50	28.57	0
A&P	br_A&P_	MSC-	0	0	6.25	81.25	6.25
		MSC+	0	7.14	64.28	28.57	0
	Fg_A&P_	MSC−	0	0	6.25	81.25	6.25
		MSC+	0	0	57.14	42.85	0
	Gp_A&P_	MSC−	0	0	0	56.25	43.75
		MSC+	0	0	0	92.85	7.14
	Co_A&P_	MSC−	0	0	0	31.25	68.75
		MSC+	0	7.14	7.14	78.57	7.14
	Si_A&P_	MSC−	0	0	0	12.5	87.5
		MSC+	0	0	0	78.57	21.42

BF area, Body Function area; A&P area, Activity and Participation area; BF_Br, Body Function_Body reaction; BF_Fg, Body Function_ Facial gesture; BF_Vr, Body Function_ Vegetative reactions; A&P_ Br, Activity and Participation_ Body reaction; A&P_Fg, Activity and Participation_ Facial gesture; A&P_Gp, Activity and Participation_Gestures and pose; A&P_Co, Activity and Participation_Contact with the outer world; A&P_Si, Activity and Participation_Speech and intonation.

## Data Availability

The data contained in this article were not published in the repository, but they are available upon request.

## Abbreviations

TBI	traumatic brain injury
DOC	disorder of consciousness
MCSURS	Minimal Conscious StateUnresponsive Conscious State
ICF	International Classification of functioning
ICD	International classification of disease
EBSCO	Elton Bryson Stephens Company
SCABL	The Bykova–Lukyanov Scale of Communication Activity
CRS-R	Coma Recovery Scale—Revised
BF	Body Function
A&P	Activity and Participation
